# Antibacterial Activity of Ethanol Extract from Australian Finger Lime

**DOI:** 10.3390/foods13152465

**Published:** 2024-08-05

**Authors:** Ruimin Zhang, Zhengyan Fan, Congyi Zhu, Yongjing Huang, Pingzhi Wu, Jiwu Zeng

**Affiliations:** Key Laboratory of South Subtropical Fruit Biology and Genetic Resource Utilization, Ministry of Agriculture and Rural Affairs, Guangdong Provincial Key Laboratory of Science and Technology Research on Fruit Tree, Institute of Fruit Tree Research, Guangdong Academy of Agricultural Sciences, Guangzhou 510640, China; xmingz@163.com (R.Z.); fanzhengyan@gdaas.cn (Z.F.); zhucongyi@gdaas.cn (C.Z.); huangyongjing@gdaas.cn (Y.H.); wupingzhi@gdaas.cn (P.W.)

**Keywords:** antibacterial effect, Australian finger lime (*Citrus australasica* L.), ethanol extract, minimum inhibitory concentration, phytochemicals

## Abstract

Australian finger lime (*Citrus australasica* L.) has become increasingly popular due to its potent antioxidant capacity and health-promoting benefits. This study aimed to determine the chemical composition, antibacterial characteristics, and mechanism of finger lime extract. The finger lime extracts were obtained from the fruit of the Australian finger lime by the ethanol extraction method. The antibacterial activity of the extract was examined by detecting the minimum inhibitory concentration (MIC) for two Gram-positive and four Gram-negative bacterial strains in vitro, as well as by assessing variations in the number of bacteria for *Candidatus* Liberibacter asiaticus (*C*Las) in vivo. GC-MS analysis was used to identify the antibacterial compounds of the extract. The antibacterial mechanisms were investigated by assessing cell permeability and membrane integrity, and the bacterial morphology was examined using scanning electron microscopy. The extract demonstrated significant antibacterial activity against *Staphylococcus aureus*, *Bacillus subtilis*, and Gram-negative bacterial species, such as *Escherichia coli*, *Agrobacterium tumefaciens*, *Xanthomonas campestris*, *Xanthomonas citri*, and *C*Las. Among the six strains evaluated in vitro, *B. subtilis* showed the highest susceptibility to the antimicrobial effects of finger lime extract. The minimum inhibitory concentration (MIC) of the extract against the tested microorganisms varied between 500 and 1000 μg/mL. In addition, the extract was proven effective in suppressing *C*Las in vivo, as indicated by the lower *C*Las titers in the treated leaves compared to the control. A total of 360 compounds, including carbohydrates (31.159%), organic acid (30.909%), alcohols (13.380%), polyphenols (5.660%), esters (3.796%), and alkaloids (0.612%), were identified in the extract. We predicted that the primary bioactive compounds responsible for the antibacterial effects of the extract were quinic acid and other polyphenols, as well as alkaloids. The morphology of the tested microbes was altered and damaged, leading to lysis of the cell wall, cell content leakage, and cell death. Based on the results, ethanol extracts from finger lime may be a fitting substitute for synthetic bactericides in food and plant protection.

## 1. Introduction

Plants generate secondary metabolites in reacting to environmental stimuli, such as herbivore attacks, abiotic stress, or interactions with other species [1] (Yang et al., 2018). These secondary metabolites provide abundant supplies of contemporary medications, chemical compounds for artificial drugs, and pharmaceutical intermediates [2,3] (Prasad et al., 2005; Elshafie et al., 2023). Exploring natural plant products for their medicinal properties is a crucial preliminary stage in developing effective drugs for various diseases. A great deal of research has proven that plant-derived products have excellent anti-inflammatory, antibacterial, antifungal, and antioxidant properties [4,5,6,7] (Yi et al., 2008; Blowman et al., 2018; Chen et al., 2018; Hassan et al., 2022). Secondary metabolites or phytochemicals, including phenols, flavonoids, alkaloids, terpenoids, and essential oils, have been demonstrated as the basis for the antimicrobial properties exhibited by plants [8,9,10] (Hwang et al., 2012; Patra, 2012; Álvarez-Martínez et al., 2021). In recent years, the application of plant extracts has received growing attention as a natural alternative to commercial synthetic chemicals in controlling primary crop diseases [7,11] (Isman, 2000; Hassan et al., 2022).

The *Citrus* genus includes lemon/limes, oranges, and mandarins. Due to their economic and nutritional values, plants of this genus are widely planted in many countries with tropical or subtropical climates, including Brazil, the United States, Japan, China, Mexico, Pakistan, and the Mediterranean [12] (Zhong & Nicolosi, 2020). Citrus fruits are rich in phytochemicals, which are bioactive compounds, such as phenolic compounds, flavonoids, vitamins, and essential oils. These phytochemicals exhibit diverse protective health benefits, including antioxidative, anti-inflammatory, antitumor, and antimicrobial activities [13,14] (Sidhic et al., 2023; Qi et al., 2023). Lime is a type of citrus fruit that has been widely cultivated across various regions. Australian finger lime (*Citrus australasica* F. Muell.) belongs to the oranges in the Rutaceae family. It mainly grows in the tropical rainforest areas of Queensland and New South Wales in Australia [15] (Johnson et al., 2022). Its fruit possesses the distinctive characteristic of oval or spherical pulp vesicles, which visually resemble the caviar in the pulp. The oval or spherical pulp vesicles give the pulp a caviar-like appearance, which makes this fruit unique to the genus *Citrus*.

Recently, worldwide interest in Australian native *Citrus* fruits has grown due to traits not found in other *Citrus* varieties [16] (Konczak and Roulle, 2011). First, finger lime is resistant to *Candidatus* Liberibacter asiaticus (*C*Las), the bacterium that causes citrus Huanglongbing (HLB) [17,18] (Alves et al., 2021; Adhikari et al., 2021). Second, the species can be used as a hybrid parent to cultivate new varieties because it produces a single embryo [19] (Dutt et al., 2021). This has opened the possibility of using it in the breeding *C*Las resistance program as it is cross-compatible with *Citrus*. Additionally, finger lime produces specific medicinal and therapeutic roles in people with weak gastrointestinal functions. This suggests that it may contain specific antibacterial components and have antibacterial activity.

Several studies that have investigated the compounds of Australian finger lime cultivars have reported the discovery of several antioxidant compounds, such as citric acid, pyrogallol, and caffeic acid [15,20] (Johnson et al., 2022; Aznar et al., 2022). However, the composition of citrus extracts varies markedly due to different cultivars, geographic locations, and extraction methods [21,22] (Droby et al., 2018; Bora et al., 2020). In addition, the differences that arise due to the variety and the extraction methods used have resulted in a shortage of comprehensive data on this subject. Moreover, to our knowledge, there are no reports regarding the antibacterial activity and mechanisms of Australian finger lime extract. Therefore, this study aimed to determine the specific chemical components of Australian finger lime extract and evaluate their selective antibacterial activity against two Gram-positive bacteria (*Staphylococcus aureus* and *Bacillus subtilis*) and five Gram-negative bacteria (*Escherichia coli*, *Agrobacterium tumefaciens*, *Xanthomonas campestris*, *Xanthomonas citri*, and *Candidatus* Liberibacter asiaticus), as well as explore their mechanisms of antibacterial action, including effects on cell permeability, membrane integrity, and bacterial morphology.

## 2. Materials and Methods

### 2.1. Plant Material and Compound Extraction

The fruits of finger lime used in this work were collected from the germplasm resources nursery of the Institute of Fruit Tree Research, Guangdong Academy of Agricultural Science (23°9′10.721″ N 113°22′17.846″ E). The finger lime cultivar evaluated in this study was Red Champagne. A voucher specimen was deposited in the −80 °C fridge of the institute under repository number AF001. The preliminary experiments used ethanol, methanol, and acetone as extraction solvents. Notably, the antibacterial efficacy of the ethanol extract (the ratio of ethanol to powder = 1:8) surpassed that of the others.

Consequently, ethanol was selected as the solvent for subsequent extractions. Furthermore, diverse ratios of dry matter to extraction solvent were evaluated in advance, revealing that the extract achieved with a ratio of 1:10 exhibited the most remarkable antibacterial effect among the other ratios of 1:5, 1:8, 1:10, 1:12, 1:15, and 1:20. Henceforth, a consistent material ratio of 1:10 was adopted for all ensuing investigations. The ripe, full fruits, including peel and pulp, were thoroughly washed after being collected, and then dried in a baking oven (Shanghai Boxun Industrial Co., Ltd., Shanghai, China) at 50 °C. After drying, the fruits were ground into a coarse powder using a blender (Tianjin Test Instrument Co., Ltd., Tianjin, China) and filtered through a 40-mesh screen. The fine powder was then stored in the refrigerator at 4 °C. A total of 10 g of powder was mixed with 90 mL of 75% ethanol. The mixture was well shaken for 2 min and kept at 50 °C for 7 h with constant agitation. After that, the extract was filtered using double layers of gauze and filter paper. The filtrate was centrifuged at 4 °C at 14,300× *g* for 10 min, and the supernatant was retained. Finally, the supernatant was obtained, lyophilized, and stored at −20 °C. Before use in the following experiments, it was sufficiently dissolved in ethanol.

### 2.2. Test Microorganisms

Pure cultures of pathogenic bacteria, including *Escherichia coli*, *Staphylococcus aureus*, *Bacillus subtilis*, *Agrobacterium tumefaciens*, and *Xanthomonas campestris*, were all purchased from the Guangdong Microbial Culture Collection Center. *Xanthomonas citri* was collected from symptomatic citrus leaves and stored in the laboratory after identification. The media used for growth was Luria–Bertani (LB) nutrient agar. The gathered organisms were cultured and preserved in LB nutrient agar slants at a temperature of 4 °C until they were required for further experimentation. The primary objective of this study was to evaluate the inhibitory effect of Australian finger lime extract on citrus canker and citrus HLB, which are both caused by Gram-negative pathogens. To maintain clarity and relevance without distracting the readers with too much detail, more Gram-negative than Gram-positive bacteria were selected in this experimental design.

### 2.3. Detection of Minimum Inhibitory Concentration (MIC)

The MIC of the extract was determined according to the turbidimetric method described by Kyhse-andersen et al. (1994) [23] and Zhang et al. (2017) [24]. A total of 100 mL of overnight-cultured and tested bacteria solution and 2.0 mL of the extract solution (the original extract) with different concentrations (e.g., 1.0, 0.5, 0.33, 0.25, 0.125, and 0.0625 mg/mL) were added to the test tubes. Then, the tubes were incubated at 37 °C for 2 h. A bacterial culture containing aseptic distilled water without extract was used as the control. Absorptions at 600 nm were measured. The lowest concentration, which inhibited the visible growth of the respective microorganisms, was taken as the MIC. Each bacteriostatic rate was calculated according to the following formula (Weng, 2010) [25]:

Bacteriostatic rate (%) = (1 − A_X_/A_0_) × 100, (1)

where A_0_ is the absorbance of the control group and A_X_ is the absorbance of the tested group. Streptomycin (concentration range, 5–100 μg/mL) was used as the positive control.

### 2.4. Antibacterial Activity of the Constituents from Finger Lime

The extract was divided into several parts using the following process to accurately determine which components had antimicrobial effects. First, the extract was added to a 3KD ultrafiltration centrifugal tube (Millipore, Bedford, MA, USA, 15 mL) and centrifuged at 5000× *g* for 2 min (we conducted multiple trials and determined that a speed of 5000× *g* and a duration of 2 min was the most appropriate to optimize efficiency and retain a small amount of liquid on the filter membrane to facilitate the washing of the material from the membrane). The extract was divided into two parts: A (>3 kDa) and B (<3 kDa). The protein and polysaccharides in Part B were separated using the ethanol precipitation process (EPP) [26,27,28] (Boulet et al., 2001; Tai et al., 2020; Robertson et al., 2007). A total of 100 μL of Part B of the extract was added to 900 μL of precooled absolute ethanol. The sample was then mixed and kept for 15 min at −20 °C. After that, it was centrifuged at 22,300× *g* for 15 min at 4 °C. The supernatant was then carefully removed into another tube, and the precipitated part was retained. Finally, Part B of the extract was separated into two components: B1 (polysaccharides and polypeptides—the precipitated portion) and B2 (low molecular weight organic matter—the supernatant). Then, 100 μL of the B1 part of the extract was added to 1000 μL of 70% ethanol. Next, the solution was mixed and kept for 30 min at 37 °C. Next, the mixture was again divided into B1-1 (polysaccharides—the precipitated portion) and B1-2 (polypeptide—the supernatant). The precipitation was dried in laminar flow and dissolved in a PBS buffer before being used for the antibacterial activity experiment. Finally, the antibacterial activity of each part of the extract was detected using the agar well diffusion method by investigating the zone of growth inhibition of the bacteria. Due to its ease of culture and preliminary experiments showing that the original extract of Australian finger lime has a good antibacterial effect in it, *E. coli* was used as the test microorganism. The bacteria were cultivated in 10 mL of LB broth at 37 °C for 24  h. The bacterial density was adjusted to 2 × 10^6^ bacteria/mL in a fresh LB broth. The culture was thoroughly blended and promptly spread onto the nutrient agar, which occupied the surface of the 90 mm-diameter Petri dishes. Subsequently, the agar was thoroughly desiccated. Wells with a diameter of five millimeters were meticulously excavated in agar using a sterile cork borer. Then, 30 μL of the sample solutions were carefully dispensed into each well. The plates were incubated at a temperature of 37 °C for 24 h. Antibacterial activity was determined by measuring the diameter of the zone of inhibition (ZOI) surrounding the well. Streptomycin was used as the positive control (10 μg/mL). The experiment was repeated three times to ensure consistent results.

### 2.5. Determination of the Anti-HLB Activity of Australian Finger Lime Extracts in the Field

#### 2.5.1. Plant Materials and Research Area

In this research, trees infected with *Candidatus* Liberibacter asiaticus (*C*Las) exhibiting classic HLB symptoms, including leaf mottling, yellowing of leaves, and dieback, were chosen for study. Quantitative PCR (qPCR) with HLBaspr-specific primers was employed to test the HLB-infected citrus trees for the presence of the *C*Las bacterium. This study was conducted in an experimental orchard planted with *Citrus reticulata* Blanco cv. Shiyue Ju trees that are five years old in Guangzhou, China (23°9′10.721″ N 113°22′17.846″ E).

#### 2.5.2. Australian Finger Lime Extract Treatment Using the Spaying Method In Vivo

The antibacterial activities of the original extract against the *C*Las bacterium were determined by measuring the *C*Las titer in the leaves of the treated branches. Kasugamycin was used as a positive control due to its importance and widespread use as an agricultural antimicrobial. The citrus branch with classic HLB symptoms and similar growth was selected, and it was sprayed with the Australian finger lime extract to wet the leaves. Before spraying, starting from the handle of the branch, the leaves on the left and right of the branch alternate leaves were picked from each side of the branch. For instance, if there were three leaves on each side and one at the top, totaling seven leaves, then starting from the handle, we picked the first on the left, second on the right, and third on the left as the initial state of this branch on Day 0. After spraying with lemon extract (original, no dilution) or kasugamycin (2000 μg/mL) for six days, we collected the remaining leaves on the branch, which were the second on the left, first on the right, and the third on the right, as the leaves after treatment. The leaf at the top was not taken. Sprayed water was used as a no-treatment control to detect the dynamic changes in *C*Las in the citrus plants under natural conditions. Three branches from two similar-growth trees were treated each time for each group.

#### 2.5.3. Genomic DNA Extraction and qPCR Analysis for the HLB Bacterium

Every individual leaf specimen was washed thrice using sterilized water. The central veins were then extracted from the leaves and chopped into fragments ranging from 1.0 to 2.0 mm. DNA was isolated from 0.2 g of fresh-weight leaf midrib tissue employing the DNeasy Plant Mini Kit by Qiagen (located in Valencia, CA, USA) with strict adherence to the instructions provided by the manufacturer. Bacterial titers were determined via the quantitative real-time PCR (qPCR) described previously (Li et al., 2008; Zhang et al., 2011 [29,30]). The qPCR was performed with specific primers and probes (Li et al., 2006): HLBasf; 50-TCGAGCGCGTATGCGAATACG-30, HLBasr; 50-AGACGGGTGAGTAACGCG-30, HLBasp; and 50-GCGTTATCCCGTAGAAAAAGGTAG-30 using a LightCycler^®^ 480 system (Roche Applied Science, Mannheim, Germany). The Ct values were recalculated into approximate bacterial counts by employing the comprehensive universal regression formula Y = 13.82 − 0.2866X, with X representing the average Ct value and Y denoting the logarithmic concentration of the target DNA copies (Li et al., 2008) [30].

### 2.6. GC-MS Chemicals and Sample Preparation

HPLC-grade methanol was purchased from Thermo Fisher Scientific Inc., and 2-chlorobenzalanine was obtained from Aladdin Reagent Co., Ltd., Shanghai, China. All other chemicals were analytical grade and were used as received. To prepare the samples, 100 μL of the stored samples at −80 °C were thawed at room temperature and transferred to 1.5 mL Eppendorf tubes, followed by the addition of 400 μL of a methanol–water solution (V:V = 4:1) and vortexing for 30 s. Ultrasonic extraction was then performed for 10 min in an ice-water bath, followed by incubation at −20 °C for 30 min. After centrifugation at 16,800× *g* for 10 min at 4 °C, 100 μL of the supernatant was transferred to glass derivatization vials, and the samples were dried using a freeze concentrator centrifugal dryer. Subsequently, 80 μL of a methoxyamine hydrochloride pyridine solution (15 mg/mL) was added to the vials, followed by vortexing for 2 min and incubation at 37 °C for 90 min for an oximation reaction. Afterward, 50 μL of BSTFA derivatization reagent (containing 1% TMCS) and 20 μL of n-hexane were added, along with 10 μL of 11 internal standards (C8/C9/C10/C12/C14/C16 at 0.16 mg/mL and C18/C20/C22/C24/C26 at 0.08 mg/mL, all in chloroform configuration). The mixture was vortexed for 2 min and reacted at 70 °C for 60 min. Finally, the samples were left at room temperature for 30 min before conducting GC-MS metabolomics analysis.

### 2.7. GC-MS Analysis

The bioactive parts of the extract (B2) were analyzed on an Agilent 7890B gas chromatography system coupled with an Agilent 5977A MSD system (Agilent Technologies Inc., Santa Clara, CA, USA). A DB-5MS fused-silica capillary column (30 m × 0.25 mm × 0.25 μm, Agilent J & W Scientific, Folsom, CA, USA) was utilized to separate the derivatives. Helium (>99.999%) was used as the carrier gas at a constant flow rate of 1 mL/min through the column. The injector temperature was maintained at 260 °C. The injection volume was 1 μL using the splitless mode. The initial oven temperature was 60 °C, which was ramped to 125 °C at a rate of 8 °C/min, to 210 °C at a rate of 4 °C/min, to 270 °C at a rate of 5 °C/min, and to 305 °C at a rate of 10 °C/min. Finally, the oven temperature was held at 305 °C for 3 min. The MS quadrupole and ion source (electron impact) temperatures were set to 150 °C and 230 °C, respectively. The collision energy was 70 eV. The active compounds were identified by matching them to the LUG database (the Untarget database of GC-MS from Lumingbio).

### 2.8. Cell Membrane Permeability

The cell membrane permeability was expressed in relative electrical conductivity according to the method described by Diao et al. (2014) [31], with slight modifications. The tested bacteria were subcultured at 30 °C and 160 rpm for 12 h, centrifuged at 3500× *g* for 10 min, and then washed with 5% glucose until the electric conductivity was near that of 5% glucose (which was the case for the isotonic bacteria). The original extract at three different concentrations (1.00 mg/mL, 0.5 mg/mL, and 0.33 mg/mL) was added to a 5% glucose solution, and the electric conductivity of the resulting mixtures was measured and recorded as L1. Additionally, different concentrations of the original extract were added into the isotonic bacteria solution, and these samples were incubated at 30 °C for 24 h. Subsequently, the electrical conductivity was marked as L2. The conductivity of the isotonic bacteria in 5% glucose (which was absent of the extract) treated in boiling water for 5 min was used as the control and marked as L0. The permeability of the membrane was calculated based on the percentage variation (V%) of the electrical conductivity according to the following formula: V% = (L2 − L1)/L0 × 100. The electric conductivities were measured using an electrical conductivity meter (DDS-11D, Shanghai Precision Science Instrument Co. Ltd., Shanghai, China).

### 2.9. Scanning Electron Microscopy (SEM)

The bacterial strains were cultured in LB broth at 160 rpm for 12 h at 37 °C. Following this, 500 μL of the extract (the original extract) was added to a 5 mL suspension of the bacteria and incubated for 6 h at a temperature of 30 °C. The resulting mixed inoculum was centrifuged at 1000× *g* for 5 min at a temperature of 4 °C. The pellet obtained was washed with a sodium phosphate buffer and fixed for 24 h at 4 °C using a solution of 2.5% glutaraldehyde in 0.1 M of sodium phosphate buffer. After three subsequent washes in the same buffer, each lasting 15 min, the samples were dehydrated in a graded ethanol (Chem-Supply) series (e.g., 30%, 50%, 70%, 90%, 95%, and 100%, each for 15 min). Following natural drying, a 20 nm-thick gold layer was applied to the samples, which were observed using a scanning electron microscope. A control group of bacterial cells not exposed to the extract underwent the same processing steps.

### 2.10. Statistical Analysis

The impact of finger lime ethanol extract at various concentrations on the radial growth of bacterial strains was assessed using a one-way analysis of variance (ANOVA). The mean differences between the treatment groups or concentration levels of the plant extracts were determined using Dunnett’s multiple comparisons test at a 5% significance level (Dunnett, 1955) [32]. ANOVA was performed using SPSS 17.0 software (SPSS Inc., Chicago, IL, USA, 2008) [33].

## 3. Results and Discussion

### 3.1. The Minimum Inhibitory Concentration (MIC) of the Extract against the Tested Bacteria In Vitro

The MIC of the extract (the original extract) against the tested bacteria was detected in this study. The extract were diluted to 500, 250, 125, and 62.5 μg/mL. The extract exhibited antibacterial activity against all test bacterial strains. The highest antibacterial rates of the extract against the studied bacterial strains ranged from 73.22% to 31.96% (Table 1). The extract had the best bacteriostatic effect on *B*. *subtilis*. The antibacterial rate decreased with a decrease in the concentration of the extract. The MIC of the extract against *A. tumefaciens*, *X. campestris*, *X. citri*, *S. aureus*, *B. subtilis*, and *E. coli* were 1000, 500, 1000, 1000, 500, and 500 μg/mL, respectively. Similar to our results, the MIC value of Mandarin (*Citrus reticulata* L.) essential oil (MEO) against *S. aureus* was found to be 500 μg/mL (Song et al., 2020) [34]. However, Li et al. (2019) [35] showed that the MIC values of finger citron essential oil (FCEO, *Citrus medica L. var. sarcodactylis*) against *S. aureus* and *B. subtilis* were 625 and 1250 μg/mL. The MIC was defined as the lowest concentration of oil inhibiting the visible growth of the bacterium being investigated. In essence, MIC represents a range of concentrations influenced by the specific dilution series. Thus, dilution methods may be one of the reasons for the differences between our results and those mentioned above by Li et al. (2019) [35]. Additionally, the difference in the antibacterial substances and the contents of the different citrus varieties may be one reason for the difference in MIC values. Some research studies have indicated that Gram-positive bacteria exhibits a greater susceptibility to the effects of essential oils compared to Gram-negative bacteria [36,37] (Mancuso et al., 2019; Lambert et al., 2001). However, no apparent differences were found in the values of the antibacterial rate and MIC between the Gram-positive strain (*S. aureus* and *B. subtilis*) and the Gram-negative strains in this work (*E. coli*, *A. tumefaciens*, *X. campestris*, and *X*. *citri*). Our study also revealed a substantial difference in the MIC between Australian finger lime extract and the positive control, streptomycin, with the former being 10–100 times higher. This variance may be attributed to the lower concentration of active ingredients in Australian finger lime extract, which further underscores the need to optimize the extraction technique for Australian finger lime to enhance its antibacterial properties.

### 3.2. Anti-HLB Activity of Finger Lime Extract In Vivo

HLB, which is triggered by *C*Las, presents an ongoing worldwide challenge and has inflicted severe damage on the citrus industry at an international level [38] (Bove, 2006). Numerous countries, including China, the United States, and Brazil, have prioritized preventing and controlling HLB, investing substantial resources in related research and development. Effective strategies against *C*Las bacterium in citrus production are still limited. Therefore, screening potential chemical compounds that are effective against *C*Las is urgently needed for the survival of the worldwide citrus industry. Although studies have shown that finger lime is resistant to *C*Las [17,39] (Ramadugu et al., 2016; Alves et al., 2021), no research has investigated the antibacterial effects of its extract against *C*Las.

Currently, *C*Las cannot be cultured in vitro, and rapid methods for testing antibacterial activity are lacking. The typical process for screening anti-HLB agents involves spraying, injecting, or soaking diseased trees in pots or fields, followed by evaluating the effects through regular observations of the symptoms or a qPCR detection of the *C*Las content [29,40] (Zhang et al., 2011; Li et al., 2016).

A spraying method was used in this study to evaluate the antibacterial effects of finger lime extract against *C*Las and its potential application in the prevention and control of *citrus* Huanglongbing. The HLB bacterium population in ShiyueJu trees was evaluated via quantitative PCR (qPCR) utilizing the HLBapsr primer set [41] (Li et al., 2006) before and after treatments. The results indicated a high titer of bacteria in the Shiyue Ju trees before the treatments, with the Ct mean values between 23.41 (≈4.8 × 10^9^ cells/g of plant tissue) and 24.33 (≈2.6 × 10^9^ cells/g of plant tissue). Six days later, after being sprayed with different chemicals, the mean Ct values improved and ranged from 24.74 (≈2.0 × 10^9^ cells/g of plant tissue) to 25.34 (≈1.4 × 10^9^ cells/g of plant tissue), which means that the titer of bacteria declined in all of the groups including the no-treatment control. The finger lime extract demonstrated a better antimicrobial activity against *C*Las when compared with Kasugamycin (reduction number of *C*Las for finger lime extract: 4.4 × 10^9^ ± 2.6 × 10^9^ cells/g of plant tissue; reduction number of *C*Las for kasugamycin: 2.3 × 10^9^ ± 5.5 × 10^8^ cells/g of plant tissue). However, there was no significant difference among the three groups (*p* > 0.05). Interestingly, while comparing the antibacterial activity ratio, the finger lime extract (0.68 ± 0.11) and kasugamycin (0.51 ± 0.00) both significantly (*p* < 0.05) reduced the *C*Las bacterial population when compared to the no-treatment control (Table 2). Finger lime extract can effectively reduce the *C*Las bacterial population. Therefore, these extracts may be used and further optimized for controlling and managing the HLB in *Citrus*. Thus, due to its natural source, finger lime extracts are not likely to result in the emergence of antibiotic-resistant bacteria. Therefore, finger lime extracts may have great value in the rescue and maintenance of citrus crops.

The leaves spaying method was used for testing the antibacterial efficacy of finger lime extract against *C*Las. Treatments included a no-treatment control, kasugamycin, and the original finger lime extract. Leaves were frozen in liquid nitrogen and ground to a fine powder for DNA isolation, followed by qPCR with *C*Las sequence-specific primers. Results are shown as the mean values with the standard error of the mean. Different letters in the same row indicate significant differences in mean values, whereas the same letter indicates insignificant differences at *p* < 0.05. Reduction in the number of *C*Las = the initial *C*Las number-6 days later *C*Las number; antibacterial activity = reduction in the number of *C*Las/initial *C*Las number. The Ct values were recalculated into approximate CLas counts using the comprehensive universal regression formula Y = 13.82 − 0.2866X.

### 3.3. Antibacterial Activity Constituents of Finger Lime Extract

**Figure 1:**
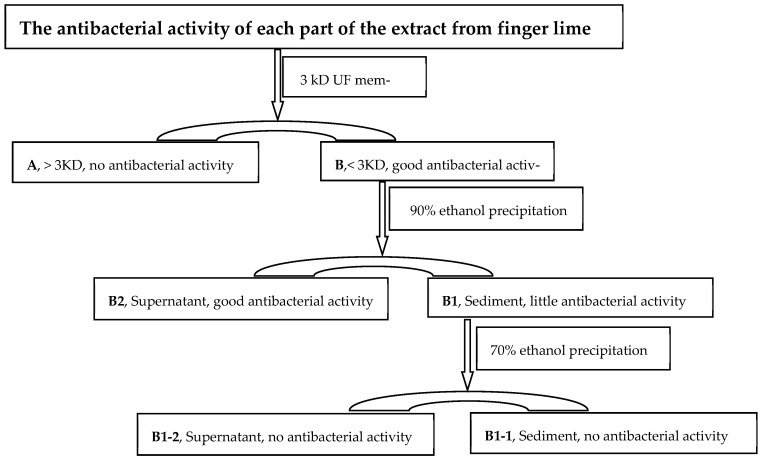
Antibacterial activity of the individual components extracted from finger lime.

*Citrus* essential oils have demonstrated various antimicrobial activities in laboratory studies [4,22,34] (Bora et al., 2020; Yi et al., 2008; Song et al., 2020). However, there is a lack of information on the antibacterial function and functional components of Australian finger lime extract. The extracts underwent a 3KD ultrafiltration centrifugal tube separation and ethanol precipitation process to identify the specific component responsible for the antibacterial activity in this work (Figure 1). *E. coli* was easy to culture. To quickly detect whether the separated original extracts had antibacterial activity and to assess their specific antibacterial effects, *E. coli* was chosen as the test bacteria strain. The portion that remained on the ultrafiltration membrane did not exhibit any noticeable antibacterial activity against *E. coli* (A part) following separation using the 3KD ultrafiltration centrifugal tube. Conversely, the filtered supernatant portion displayed significant antibacterial activity (Part B), demonstrating efficacy comparable to that of the original extract (the diameter of the inhibition zone was 12.32 mm). Protein was precipitated with >90% ethanol at all temperatures [26] (Robertson et al., 2007). After adding precooled ethanol to Part B of the extract mentioned above (resulting in a final ethanol concentration of 90%), Part B was divided into B1 and B2. The B2 part (supernatant) exhibited excellent antibacterial activity against *E. coli*, while the B1 (sediment) part showed minimal antibacterial activity (Table 3). When the B1 part was mixed with a specific ethanol concentration to achieve a final ethanol concentration of 70%, it was further divided into B1-1 and B1-2. The results indicated that both B1-1 and B1-2 had almost no antibacterial activity (Table 3). The B2 part was used for further GC-MS analysis based on the antibacterial activity results.

### 3.4. GC-MS Analysis

The compounds of the extract (the B2 part of the extract) was analyzed using GC-MS. The results revealed that 360 components were present in that sample (Supplementary Figure S7 and Table 1). Among the main components, the substances found were carbohydrates (31.159%), organic acid (30.909%), alcohols (13.380%), polyphenols (5.660%), esters (3.796%), and alkaloids (0.612%) (Table 4). The primary organic acids of the extract identified were aliphatic acids (28.086%), alicyclic acids (2.500%), and aromatic acids (0.323%). The major parts in the aliphatic acid content were proline (4.931%), 4-hydroxybutyric acid (2.375%), L-alanine (1.979%), succinic acid (1.793%), N-methylglutamic acid (1.427%), and L-aspartic acid (1.324%). Forty-seven kinds of carbohydrates were found in the extract, where D-tagatose (4.372%), ethyl beta-d-glucopyranoside (2.566%), melezitose (2.558%), rhamnose (2.542%), glucose (2.258%), trisaccharide (2.223%), 1-ketose (2.083%), ribopyranose (1.989%), galactose (1.861%), and glucosamine (1.343%) were the major compounds. Fifteen kinds of alcohols were discovered in the extract, with the main components being myoinositol (4.129%), glycerol (2.835%), lactitol (1.157%), delta-tocopherol (1.076%), and phytol (0.878%). Nineteen kinds of polyphenols were examined. The polyphenols exhibited quinic acid (4.098%) as the primary compound, followed by 4-hydroxycinnamic acid (0.415%), coniferin (0.347%), sinapic acid (0.294%), chlorogenic acid (0.214%), and piceatannol (0.098%). The alkaloids were only 0.612% of the extract, with maleimide (0.128%) being the most abundant.

Up to the top 10 most abundant compounds in each category are listed. For a detailed list of compounds, as well as the GC-MS chromatogram and resultant mass peaks of the ethanol extract of finger lime, please refer to Table S1 and Figure S6 in the Supplementary Material.

Our results show that the antibacterial properties of the extract may be attributed to the advantageous impact of phenolic acid (5.571%), flavonoid (0.089%), and alkaloid (0.612%) compounds. Many polyphenols, flavonoids, and alkaloids in plants have been proven to possess excellent antimicrobial activities against a diverse range of bacteria, such as *Streptococcus sanguinis*, *Candida albicans*, and *E. coli* [42,43,44] (Barbieri et al., 2017; Lin et al., 2019; Adamczak et al., 2020). In this study, we discovered an array of polyphenols possessing antibacterial properties, such as quinic acids, 4-hydroxycinnamic, chlorogenic acid, malic acid, and citric acid, against many bacteria, including *S. aureus* and *E. coli* [16,45,46] (Bai et al., 2018; Netzel et al., 2007; Konczak et al., 2011). Gallocatechin (belonging to flavonoids) and trigonelline (belonging to alkaloids), which were also found in the finger lime extracts in this study, has been proven to exert a robust antibacterial effect that is assayed against many bacteria and their isolated strains, such as *E. coli*, *Pseudomonas aeruginosa*, and *Proteus mirabilis* [47,48,49] (Servillo et al., 2011; Zheng et al., 2016; Anwar et al., 2018).

Additionally, there were some other compounds found in this work, such as phytol (0.878%), aconitic acid (0.898%), and glycolic acid (0.688%), which also have potential antibacterial activity [50,51,52] (Saha et al., 2020; Fernandes et al., 2020; Bruni & Klasson, 2022). Therefore, this study also suggests that the antibacterial activities of the extract from finger lime were due to several compounds, not only due to phenolics, flavonoids, and alkaloids. Furthermore, the minor constituents in the extracts may also play a role in their antibacterial properties, potentially through a synergistic interaction with other bioactive compounds.

However, the specific chemicals detected in this study differed from Johnson et al. (2022) [15], who studied the comparative chemical composition of current commercially available Australian finger lime cultivars. Hydroxybenzoic, hydroxycinnamic, and chlorogenic acids were the main phenolic acids discovered in Australian finger lime cultivars (Johnson et al., 2022) [15]. In that study, quinic acid, 4-hydroxycinnamic acid, and coniferin were the main phenolic acids, with chlorogenic acid as the fifth most predominant. In addition, they found many terpenoids, especially monoterpene, in the finger lime peels (Johnson et al., 2022) [15]. In contrast, we found only one kind of diterpenoid (phytol) and two triterpenoids (squalene and lanosterol). The quantities and types of substances extracted depend on the extraction conditions, solvent, and pH (Chen et al., 2018) [6]. In addition, we narrowed down the range of components with antimicrobial functions in this study by subjecting the extract to several screening processes. Thus, we predicted these differences might be due to different extraction solutions and methods.

### 3.5. Cell Membrane Permeability

**Figure 2:**
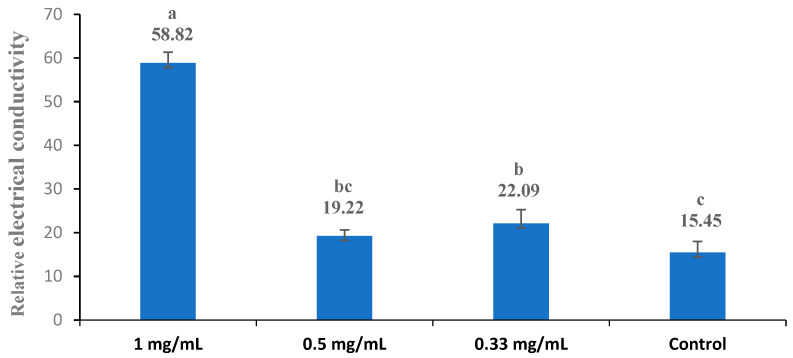
Effect of different concentrations of the finger lime extracts on the membrane permeability of *B. subtilis*. Cells in the logarithmic growth phase were treated with various (1, 0.5, and 0.33 mg/mL) doses of finger lime extract for 24 h. The membrane permeability was determined as described in the Section 2. The conductivity of the isotonic bacteria in 5% glucose (which was absent of the extract) treated in boiling water for 5 min was used as the control. The values represent the means of the three independent replicates; error bars indicate the SD. According to Duncan’s test, the means followed by the same letter above bars were not significantly different at the 0.05 probability level.

**Figure 3:**
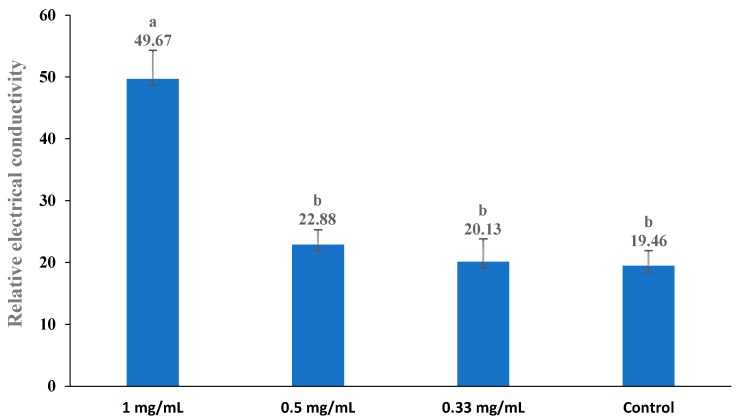
Effect of different concentrations of finger lime extracts on the membrane permeability of *E. coli*. Cells in the logarithmic growth phase were treated with various (1, 0.5, and 0.33 mg/mL) doses of finger lime extract for 24 h. The membrane permeability was determined as described in the Section 2. The conductivity of the isotonic bacteria in 5% glucose (which was absent of the extract) treated in boiling water for 5 min was used as the control. The values represent the means of three independent replicates; error bars indicate the SD. According to Duncan’s test, the means followed by the same letter above bars were not significantly different at the 0.05 probability level.

**Figure 4:**
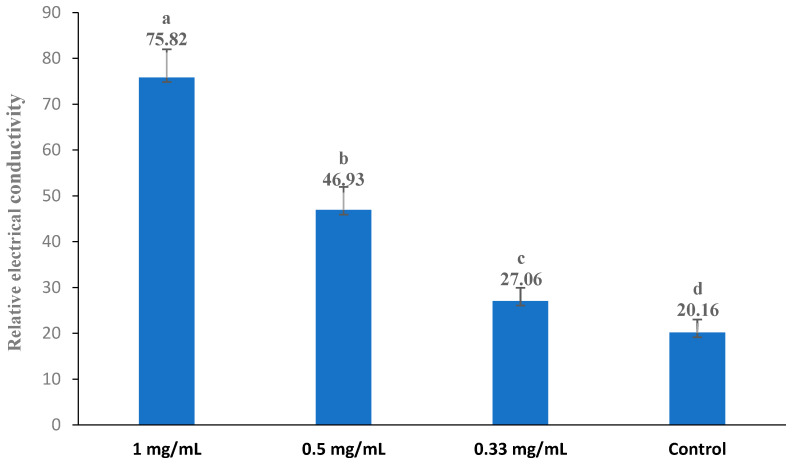
Effect of different concentrations of the finger lime extracts on the membrane permeability of *X. citri*. Cells in the logarithmic growth phase were treated with various (1, 0.5, and 0.33 mg/mL) doses of finger lime extract for 24 h. The membrane permeability was determined as described in the Section 2. The conductivity of the isotonic bacteria in 5% glucose (absent of the extract) treated in boiling water for 5 min was used as the control. The values represent the means of three independent replicates; error bars indicate the SD. According to Duncan’s test, the means followed by the same letter above bars were not significantly different at the 0.05 probability level.

**Figure 5:**
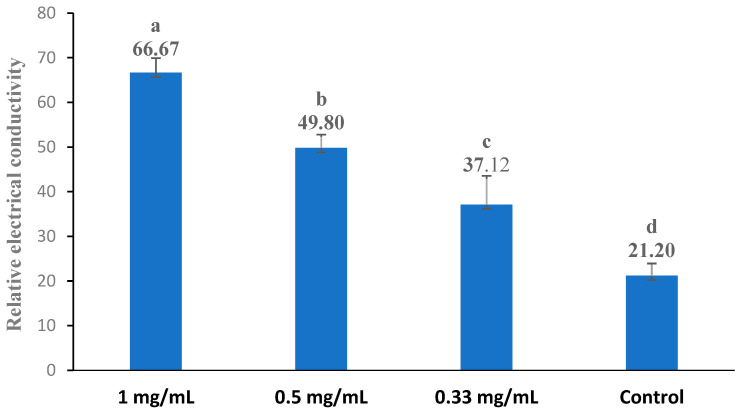
Effect of different concentrations of the finger lime extracts on the membrane permeability of *X. campestris*. Cells in the logarithmic growth phase were treated with various (1, 0.5, and 0.33 mg/mL) doses of finger lime extract for 24 h. The membrane permeability was determined as described in the Section 2. The conductivity of the isotonic bacteria in 5% glucose (which was absent of the extract) treated in boiling water for 5 min was used as the control. The values represent the means of three independent replicates; error bars indicate the SD. According to Duncan’s test, the means followed by the same letter above bars were not significantly different at the 0.05 probability level.

**Figure 6:**
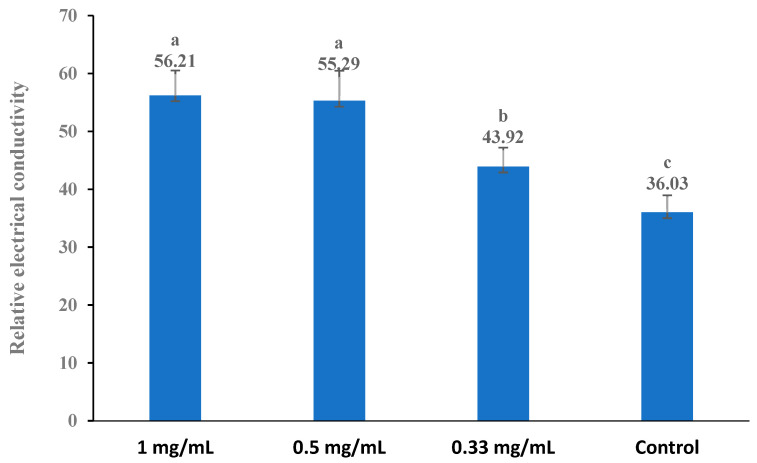
Effect of different concentrations of the finger lime extracts on the membrane permeability of *A. tumefaciens*. Cells in the logarithmic growth phase were treated with various (1, 0.5, and 0.33 mg/mL) doses of finger lime extract for 24 h. The membrane permeability was determined as described in the Section 2. The conductivity of the isotonic bacteria in 5% glucose (which was absent of the extract) treated in boiling water for 5 min was used as the control. The values represent the means of three independent replicates; error bars indicate the SD. According to Duncan’s test, the means followed by the same letter above bars were not significantly different at the 0.05 probability level.

The bacterial cell membrane is a thin, hydrophobic layer that serves as a physical barrier, effectively separating the aqueous cytoplasm from the external environment or the interiors of other cellular compartments (Nakae 1986) [53]. The change in the conductivity of the bacterial suspension reflected the difference in the permeability of the cell membrane. The influence of the extract (the original extract) on the cell membrane permeability of bacteria is shown in Figure 2, Figure 3, Figure 4, Figure 5 and Figure 6. Compared with the control, applying the extract at the highest concentrations (1 mg/mL) resulted in a significant increase in the permeability for all test bacteria. The high extract concentration resulted in significantly higher permeability values in most test bacteria strains, except *B. subtilis* and *E. coli*. In summary, the relative electric conductivity of the test bacterial suspension, meaning that the permeability of the bacterial membrane would increase correspondingly, caused the leakage of cell contents and led to cell death. These results imply that the finger lime extracts altered the permeability of the membrane, and at least one of the mechanisms of action of this extract is related to the disorders in the cell membranes of bacteria.

### 3.6. Effects of Antibacterials on the Bacterial Cell Wall

**Figure 7:**
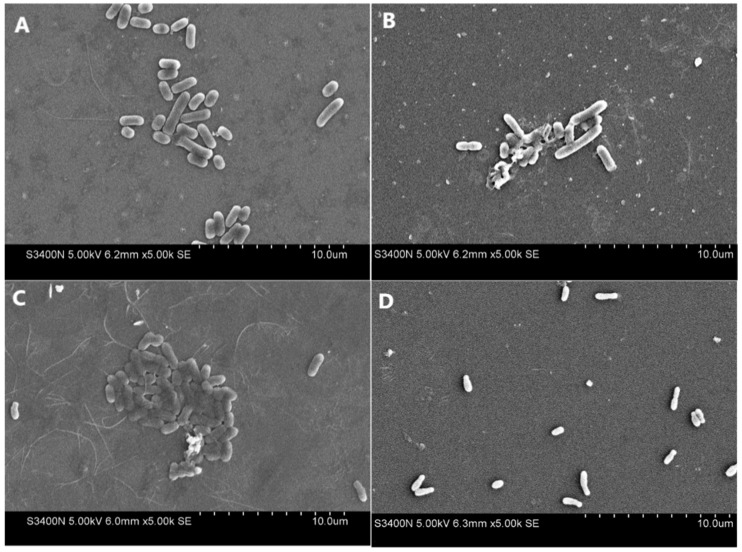

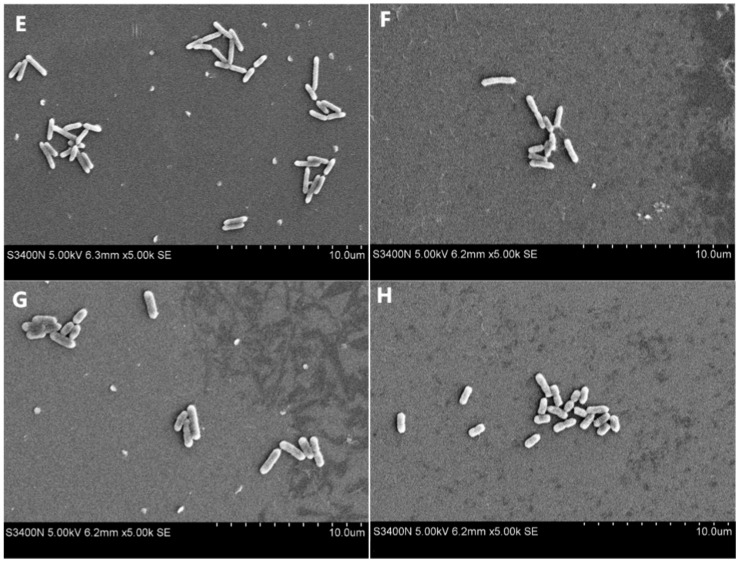
Scanning electron microscope observations of the four Gram-negative bacteria ((**A**,**B**) for *E. coli*, (**C**,**D**) for *A. tumefaciens*, (**E**,**F**) for *X. campestris*, and (**G**,**H**) for *X. citri*) after 6 h of incubation at 30 °C in the absence (**A**,**C**,**E**,**G**) and presence (**B**,**D**,**F**,**H**) of finger lime extract. The treated concentration of the finger lime extracts was 1 mg/mL of the original extract.

**Figure 8:**
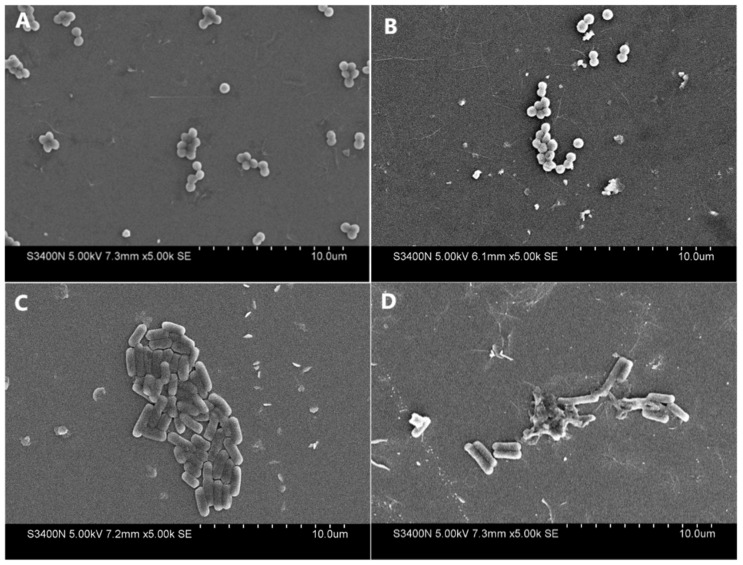
Scanning electron microscope observations of two Gram-positive bacteria ((**A**,**B**) for *S. aureus* and (**C**,**D**) for *B. subtilis*) after 6 h of incubation at 30 °C in the absence (**A**,**C**) and presence (**B**,**D**) of finger lime extract. The treated concentration of the finger lime extracts was 1 mg/mL of the original extract.

The treated bacteria were observed using SEM to further investigate the morphology of the bacteria in response to the extract (the original extract). The electron micrographs of both the untreated (control) and extract-treated microbial cells are presented in Figure 7 and Figure 8. The figures reveal the normal cell structures, shapes, and smooth surfaces in the control group compared with the altered structures observed in the treated groups. The extract was able to disrupt the cell structures and destabilize all the tested microbes in the treated group. The bacterial cells of all tested microbes in the treated group underwent different levels of distinct morphological alterations and lysis of membrane integrity, especially *A. tumefaciens* (Figure 7). This may explain why *A. tumefaciens* was more sensitive to the extract compared to most of the other tested microbes. Furthermore, the treated groups exhibited stacked cells, potentially resulting in changes and deformities, as well as causing them to deviate from their original morphology compared to the control cells (O’Donovan & Brooker 2001) [54].

The effects of antibacterial agents on the morphology of bacterial cells exhibited diverse outcomes, including the separation of the cytoplasmic membrane from the cell wall, leakage of cytoplasmic content, lysis of the cell, and distortion of the cell (Shen et al., 2015; Teng et al., 2014) [55,56]. Our results suggested that the finger lime extracts damaged the cell structure, promoting the uptake of phenolics and other components into the cells and leading to the leaking of the protoplasm and lysis of the outer membrane, thus resulting in cell death. Studies have found that morphological abnormalities and the loss of cell content can cause cell death, which supports our results (Shin et al., 2007; Chen et al., 2018) [6,57]. Previous studies have also proven that quinic acid (the most abundant phenolic acid in our extract) and chlorogenic acid could change glycerophospholipid and fatty acid levels to interfere with membrane fluidity. This feature affected the normal functions of the potassium and calcium channels, damaged the cell membrane, and finally penetrated the cell to inhibit cell wall synthesis and cell division (Bai et al., 2022; Bai et al., 2018) [46,58]. These findings suggest that at least two phenolic components (quinic acid and chlorogenic acid) were part of the antibacterial activity components of the extract.

## 4. Conclusions

This study first clearly proved the inhibitory effects of finger lime extract on both Gram-positive and Gram-negative bacteria. It demonstrated that there were 360 compounds in the finger lime extracts. Quinic acid and other polyphenols were predicted to be the main components of the antibacterial activity of the extracts. Additionally, finger lime extract could destroy the integrity of bacterial cell walls and increase the permeability of the cell membrane, leading to the leakage of intracellular components into the external environment. The above findings suggest that finger lime’s ethanol extract exhibits potential as a substitute for synthetic bactericides in food and plant protection, especially *Candidatus* Liberibacter asiaticus (*C*Las), which causes Huanglongbing. Nonetheless, a deeper investigation into the molecular pathways involved in these processes is essential.

## Figures and Tables

**Table 1:** Antibacterial activity of finger lime extract against selected pathogenic bacteria.

Bacteria	The Antibacterial Rates at Each Concentration (%)						MIC (μg/mL)	
	1000 μg/mL	500 μg/mL	330 μg/mL	250 μg/mL	125 μg/mL	62.5 μg/mL	Finger Lime	Streptomycin
*Agrobacterium tumefaciens*	60.17	23.81	5.14	1.22	0	0	1000	100
*Xanthomonas campestris*	73.22	41.20	19.43	10.28	3.57	0.82	500	5
*Xanthomonas citri*	31.96	14.87	6.43	1.74	0	0	1000	10
*Staphylococcus aureus*	32.03	15.37	5.75	1.59	0	0	1000	15
*Bacillus subtilis*	53.12	29.69	6.58	0	0	0	500	5
*Escherichia coli*	57.42	34.05	10.27	7.22	1.44	0	500	5

**Table 2:** Antibacterial efficacy of finger lime extract against *Ca*. L. asiaticus (*C*Las).

	Initial Ct	6 Days Later Ct	Reduction in the Number of *C*Las (Cells/g)	Antibacterial Activity Ratio
Finger lime extract	23.41 ± 0.85 a	25.34 ± 1.23 a	4.4 × 10^9^ ± 2.6 × 10^9^ a	0.68 ± 0.11 a
Kasugamycin	24.33 ± 0.78 a	24.67 ± 0.73 a	2.3 × 10^9^ ± 5.5 × 10^8^ a	0.51 ± 0.00 a
No-treatment control	23.64 ± 0.34 a	24.74 ± 0.33 a	7.7 × 10^8^ ± 4.5 × 10^8^ a	0.20 ± 0.04 b

For each study, different letters in the same column indicate significant differences in the mean values at *p* ≤ 0.05, while the same letter indicates insignificant differences.

**Table 3:** Antibacterial efficacy of the different fractions obtained from the finger lime extracts against *E. coli*.

	The Original Extract 2500 μg/mL	A 2500 μg/mL	B 2500 μg/mL	B1 2500 μg/mL	B2 2500 μg/mL	B1-1 2500 μg/mL	B1-2 2500 μg/mL	Streptomycin 10 μg/mL
Diameter of the Inhibition Zone (mm)	12.32	0.00	12	1.2	11	0.5	0.2	8.00

**Table 4:** Chemical composition of the extracts from finger lime.

Retention Time (min.)	Compound	Relative Area (%)
Organic Acids		
Aliphatic organic acids		
7.222	L-alanine	1.979
8.518	N-methylglutamic acid	1.427
8.953	4-hydroxybutyric acid	2.375
9.159	L-valine	0.809
10.708	Proline	4.931
11.037	Succinic acid	1.793
11.929	Serine	0.905
15.375	L-aspartic acid	1.324
15.547	Glucosaminic acid	0.9
20.279	Aconitic acid	0.898
	...	
28.086		
Alicyclic organic acids		
5.826	Shikimic acid	0.039
7.779	2-furoic acid	0.03
9.337	4-hydroxyproline	0.006
12.948	Glycyl proline	0.009
13.364	Tranexamic acid	0.113
14.845	Cholic acid	0.028
14.974	Glycyl tyrosine	0.004
15.309	Oxoproline	1.954
20.705	Lactobionic acid	0.225
24.684	Digalacturonic acid	0.086
	...	
2.500		
Aromatic acids		
16.027	Dl-dopa	0.036
16.461	N-acetyl-d-tryptophan	0.016
17.548	L-phenylalanine	0.271
0.323		
Carbohydrates		
14.85	Glucosamine	1.343
22.868	D-tagatose	4.372
23.302	Galactose	1.861
23.68	Glucose	2.258
24.387	Ribopyranose	1.989
24.444	Melezitose	2.558
24.86	1-kestose	2.083
28.14	Rhamnose	2.542
28.173	Ethyl beta-d-glucopyranoside	2.566
33.331	Trisaccharide	2.223
	...	
31.159		
Alkaloids		
7.291	1-butylamine	0.046
7.705	Maleimide	0.128
7.925	Piperidone	0.024
10.65	Niacinamide	0.1
11.53	1-methylhydantoin	0.014
13.832	5-methoxytryptamine	0.04
13.971	Cyclohexylamine	0.018
14.511	Serotonin	0.012
24.473	Trigonelline	0.113
30.762	N-acetyl-5-hydroxytryptamine	0.109
	...	
0.612		
Polyphenols		
Phenolic acids		
5.826	Shikimic acid	0.039
15.926	Cinnamic acid	0.005
16.005	3,4-dihydroxycinnamic acid	0.017
22.526	Chlorogenic acid	0.214
22.61	Quinic acid	4.098
24.112	4-hydroxycinnamic acid	0.415
25.972	Coniferin	0.347
27.991	Piceatannol	0.098
28.192	4-methoxycinnamic acid	0.04
28.829	Sinapic acid	0.294
	...	
5.571		
Flavonoids		
11.848	Epicatechin	0.001
14.743	Formononetin	0.003
15.678	Arbutin	0.015
17.184	Catechin	0.001
20.604	Gallocatechin	0.038
33.871	Daidzein	0.023
34.716	Epigallocatechin	0.008
0.089		
Alcohols		
10.242	Glycerol	2.835
11.168	Phytol	0.878
18.099	Galactitol	0.243
18.099	3-deoxyhexitol	0.243
23.67	Lactitol	1.157
25.286	Galactinol	0.555
26.755	Myo-inositol	4.129
30.601	2-methylpropan-2-ol	0.49
31.909	Prenol	1.076
35.648	Delta-tocopherol	1.076
	...	
13.380		
Esters		
20.537	Glycerol 3-phosphate	0.458
20.963	Inosine-5′-monophosphate	0.213
24.268	Glucaric acid gama-lactone	0.337
27.204	Myristyl myristate	0.415
33.826	Beta-mannosylglycerate	2.373
3.796		
Others		
24.421	D-erythro-sphingosine	2.702
5.068	Methylamine	0.897
10.091	Ethanolamine	0.539
12.958	Putrescine	0.335
25.826	Phytosphingosine	0.123
27.263	Guanine	0.019
15.975	5-hydroxynorvaline	0.009
	...	
14.484		

## Data Availability

The original contributions presented in the study are included in the article/Supplementary Material, further inquiries can be directed to the corresponding author.

## Supplementary Materials

The following supporting information can be downloaded at: https://www.mdpi.com/article/10.3390/foods13152465/s1, Figure S1. Inhibitions zone of finger lime extracts against *Escherichia coli* at multiple concentrations (0.25, 0.29, 0.33, 0.4, 0.5, 0.67, 1 mg/mL). Figure S2. Inhibitions zone of finger lime extracts against *Agrobacterium tumefaciens* at multiple concentrations (0.25, 0.29, 0.33, 0.4, 0.5, 0.67, 1 mg/mL). Figure S3. Inhibitions zone of finger lime extracts against *Staphylococcus aureus* at multiple concentrations (0.33, 0.4, 0.5, 0.67, 1 mg/mL). Figure S4. Inhibitions zone of finger lime extracts against *Bacillus subtilis* at multiple concentrations (0.25, 0.29, 0.33, 0.4, 0.5, 0.67, 1 mg/mL). Figure S5. Inhibitions zone of finger lime extracts against *Xanthomonas citri* at multiple concentrations (0.33, 0.4, 0.5, 0.67, 1 mg/mL). Figure S6. Inhibitions zone of finger lime extracts against *Xanthomonas campestris* at multiple concentrations (0.33, 0.4, 0.5, 0.67, 1 mg/mL). Figure S7. GC-MS Chromatogram and the resultant mass peak of the ethanol extract of finger lime. Table S1. Chemical composition of the extracts from finger lime.
