# Supplementary material for: Antibacterial Activity of Ethanol Extract from Australian Finger Lime
Source: Foods. 2024 Aug 5;13(15):2465. doi: 10.3390/foods13152465 (PMC11311350; doi:10.3390/foods13152465)
Supplement: Supplementary file 1 [file foods-13-02465-s001.zip › foods-3121877-supplementary.pdf]

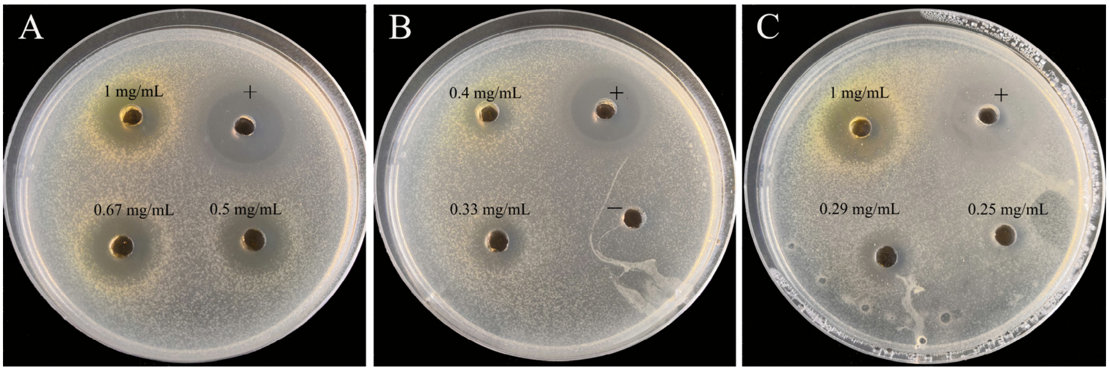

Figure S1. Inhibitions zone of finger lime extracts against *Escherichia coli* at multiple concentrations (0.25,0.29,0.33,0.4,0.5,0.67,1mg/mL). Penicillin-streptomycin was used as a positive control (+),while the distilled water was used as a negative control（-）.

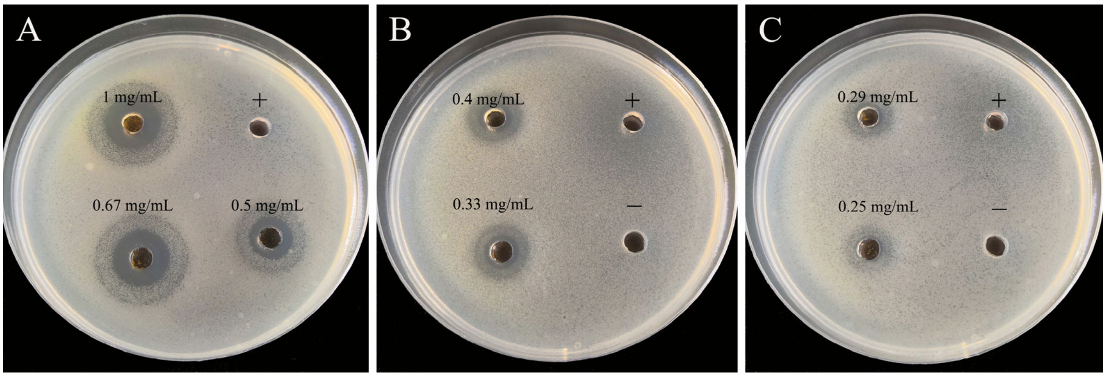

Figure S2. Inhibitions zone of finger lime extracts against *Agrobacterium tumefaciens* at multiple concentrations (0.25,0.29,0.33,0.4,0.5,0.67,1mg/mL). Penicillin-streptomycin was used as a positive control (+),while the distilled water was used as a negative control（-）.

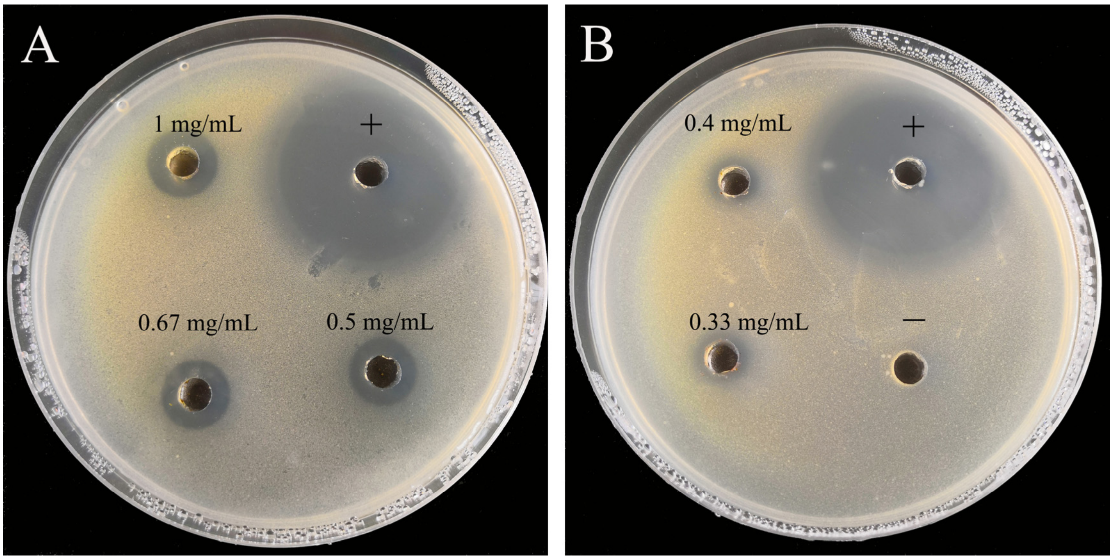

Figure S3. Inhibitions zone of finger lime extracts against *Staphylococcus aureus* at multiple concentrations (0.33,0.4,0.5,0.67,1mg/mL). Penicillin-streptomycin was used as a positive

control (+),while the distilled water was used as a negative control（-）.

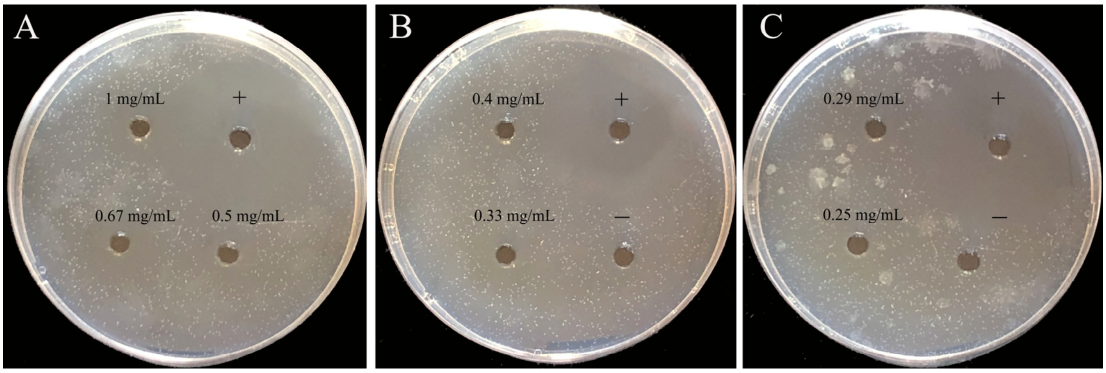

Figure S4. Inhibitions zone of finger lime extracts against *Bacillus subtilis* at multiple concentrations (0.25,0.29,0.33,0.4,0.5,0.67,1mg/mL). Penicillin-streptomycin was used as a positive control (+),while the distilled water was used as a negative control（-）.

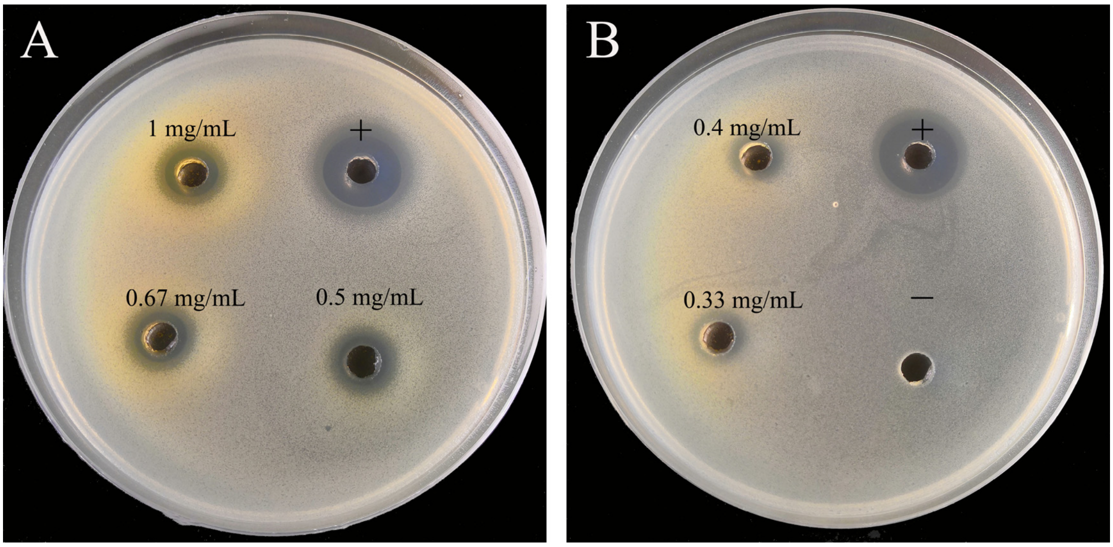

Figure S5. Inhibitions zone of finger lime extracts against *Xanthomonas citri* at multiple concentrations (0.33,0.4,0.5,0.67,1mg/mL). Penicillin-streptomycin was used as a positive control (+), while the distilled water was used as a negative control（-）.

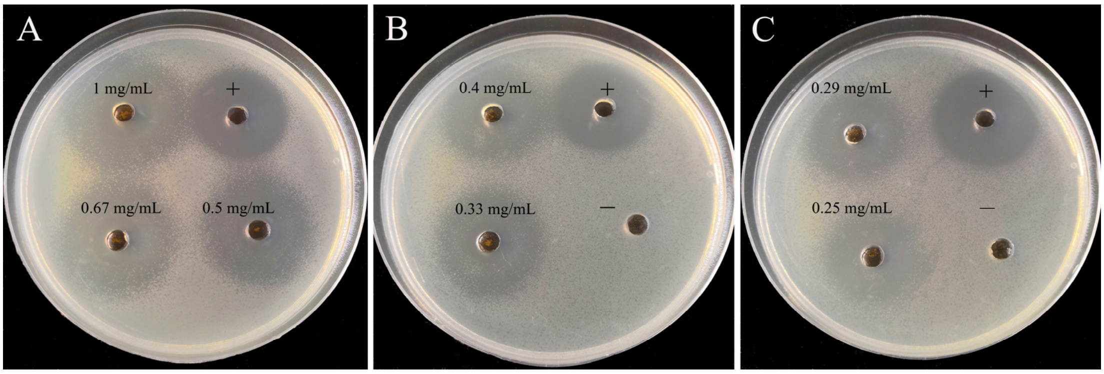

Figure S6. Inhibitions zone of finger lime extracts against *Xanthomonas campestris* at multiple concentrations (0.33,0.4,0.5,0.67,1mg/mL). Penicillin-streptomycin was used as a

positive control (+), while the distilled water was used as a negative control（-）.

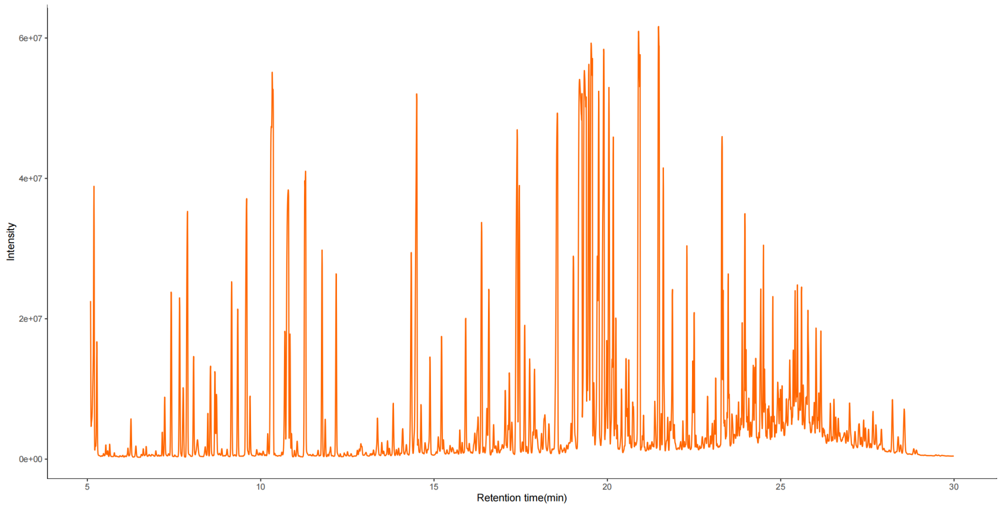

Figure S7. GC-MS Chromatogram and the resultant mass peak of the ethanol extract of finger lime. Instrumentation: Agilent 7890B gas chromatography, injected 1 μL (260°C) sample, column: 30 m × 0.25 mm × 0.25 μm film thickness (DB5MS), temperature program: initial 60°C, ramped to 125°C at a rate of 8°C/min, to 210°C at a rate of 4°C/min, to 270°C at a rate of 5°C/min, to 305°C at a rate of 10°C/min (3min), detection: Agilent 5977A MSD system (Agilent Technologies Inc., CA, USA).

Table S1. Chemical composition of the extracts from finger lime.

Retention Time (min.)	Compound	Relative area (%)
	**Organic acids**	
	**Aliphatic organic acids**	
5.108	2-ketoadipic acid	0.163
5.493	2-hydroxypentanoic acid	0.009
6.334	Pyruvic acid	0.027
6.505	L-lactic acid	0.154
6.627	Linoleic acid	0.024
6.775	Glycolic acid	0.688
7.222	L-alanine	1.979
7.372	Alpha-aminoadipic acid	0.010
7.474	Sarcosine	0.030
7.947	3-hydroxypropionic acid	0.361
8.294	L-methionine	0.032
8.371	Oleic acid	0.007
8.401	N-methylalanine	0.026
8.518	N-methylglutamic acid	1.427
8.584	Ornithine	0.016
8.953	4-hydroxybutyric acid	2.375
9.159	L-valine	0.809
9.177	Lysine	0.005
9.387	Dodecanoic acid	0.103
9.665	3-aminoisobutyric acid	0.002
9.726	Acetoacetic acid	0.155
9.752	Ethylsuccinate	0.012
10.073	Caprylic acid	0.020

10.318	L-leucine	0.002
10.393	Guanidinosuccinate	0.024
10.618	L-isoleucine	0.435
10.708	Proline	4.931
10.851	Glycine	0.313
11.037	Succinic acid	1.793
11.037	L-2-hydroxyglutaric acid	0.280
11.262	Isolinoleic acid	0.003
11.32	Glyceric acid	0.177
11.562	Itaconic acid	0.157
11.694	Citraconic acid	0.015
11.746	L-threonic acid	0.004
11.753	Fumaric acid	0.068
11.929	Serine	0.905
12.132	Cyano-l-alanine	0.034
12.291	L-cysteine	0.001
12.445	L-threonine	0.206
12.636	Glutaric acid	0.203
12.808	Saccharopine	0.011
13.334	Beta-alanine	0.024
13.409	2-deoxytetronic acid	0.334
13.518	Glycocyamine	0.013
13.609	Isocitric acid	0.112
13.616	N-acetylglutamate	0.034
13.738	Beta-hydroxymyristic acid	0.382
13.81	L-cystathionine	0.002
13.832	Capric acid	0.039
13.854	Isopentadecanoic acid	0.000
13.869	5-aminovaleric acid	0.020
13.966	N-acetylornithine	0.180
14.37	Citrulline	0.027
14.506	Beta-glutamic acid	0.029
14.722	Malate	0.524
14.724	3-phosphoglycerate	0.088
14.937	L-asparagine	0.162
15.286	Glutamyl-valine	0.004
15.308	N-carbamylglutamate	0.160
15.375	L-aspartic acid	1.324
15.544	Gamma-aminobutyric acid	0.376
15.547	Glucosaminic acid	0.900
15.708	Cerotinic acid	0.047
15.754	L-glutamine	0.030
16.084	Malonic acid	0.230
16.084	Oxalacetic acid	0.227
16.201	Erythronic acid	0.559
16.201	Dihydroxymalonic acid	0.562
17.095	3-hydroxy-3-methylglutaric acid	0.062
17.491	L-glutamic acid	0.110
19.699	Homocystine	0.004
19.954	Nonanoic acid	0.037
20.279	Aconitic acid	0.898
20.502	Xylonic acid	0.040
20.537	Glycerol 3-phosphate	0.458
20.564	6-phosphogluconic acid	0.166
21.067	Glutathione	0.239
21.382	3-epicholic acid	0.119
21.617	Montanic acid	0.054

22.211	Citric acid	0.388
24.158	Galacturonic acid	0.191
25.043	Pantothenic acid	0.036
25.199	Gluconic acid	0.173
25.2	Galactonic acid	0.082
25.376	Galactaric acid	0.465
25.385	Tartaric acid	0.044
27.508	2-aminoheptanedioic acid	0.132
	**28.086**	
	**Alicyclic organic acids**	
5.826	Shikimic acid	0.039
7.779	2-furoic acid	0.030
9.337	4-hydroxyproline	0.006
9.957	L-histidine	0.002
12.948	Glycyl proline	0.009
13.364	Tranexamic acid	0.113
14.095	Chenodeoxycholic acid	0.002
14.102	Deoxycholic acid	0.000
14.845	Cholic acid	0.028
14.974	Glycyl tyrosine	0.004
15.309	Oxoproline	1.954
20.705	Lactobionic acid	0.225
24.684	Digalacturonic acid	0.086
	**2.500**	
	**Aromatic acids**	
16.027	Dl-dopa	0.036
16.461	N-acetyl-d-tryptophan	0.016
17.548	L-phenylalanine	0.271
	**0.323**	
	**Carbohydrates**	
10.225	Erythrose	0.916
10.354	Glucose-6-phosphate	0.005
11.694	Glucose-1-phosphate	0.014
14.085	D-fructose-1-phosphate	0.001
14.11	D-ribose-5-phosphate	0.005
14.85	Glucosamine	1.343
16.763	N-acetylgalactosamine	0.009
16.82	D-fructose-6-phosphate	0.017
17.251	Lactulose	0.001
17.569	N-acetyl-d-mannosamine	0.002
17.625	Cellobiose	0.014
18.264	D-arabinose	0.151
18.267	D-ribose	0.177
18.638	Trehalose-6-phosphate	0.018
18.703	D-xylulose	0.052
19.56	D-fucose	0.112
19.91	Isomaltose	0.169
20.283	3,6-anhydro-d-galactose	0.667
20.705	Xylofuranose	0.225
22.285	D-myo-inositol 4-phosphate	0.081
22.35	Melibiose	0.309
22.394	Sophorose	0.318
22.868	D-tagatose	4.372
22.965	D-fructose	0.084
23.15	N-acetyl-d-hexosamine	0.395
23.302	Galactose	1.861
23.559	Gluconic acid lactone	0.331

23.68	Glucose	2.258
23.777	D-fructose-1,6-bisphosphate	0.018
24.387	Ribopyranose	1.989
24.444	Melezitose	2.558
24.523	Methyl 6-deoxy-alpha-l-galactofuranoside	0.053
24.746	Arabinofuranose	0.167
24.86	1-kestose	2.083
24.877	Maltotriose	0.659
25.286	Galactinol	0.555
25.519	Methyl 6-deoxy-alpha-l-galactopyranoside	0.117
26.389	Beta-gentiobiose	0.012
26.602	N-acetyl-d-glucosamine	0.075
27.469	Talose	0.171
27.86	Mannobiose	0.024
28.14	Rhamnose	2.542
28.173	Ethyl beta-d-glucopyranoside	2.566
29.424	Methyl beta-d-glucopyranoside	0.446
29.669	D-xylose	0.934
33.331	Trisaccharide	2.223
38.227	Raffinose	0.058
	**31.159**	
	**Alkaloids**	
7.291	1-butylamine	0.046
7.705	Maleimide	0.128
7.925	Piperidone	0.024
8.414	Anabasine	0.003
8.998	Hypoxanthine	0.004
10.65	Niacinamide	0.100
11.53	1-methylhydantoin	0.014
12.68	Adenine	0.001
13.832	5-methoxytryptamine	0.040
13.971	Cyclohexylamine	0.018
14.511	Serotonin	0.012
24.473	Trigonelline	0.113
30.762	N-acetyl-5-hydroxytryptamine	0.109
	**0.612**	
	**Polyphenols**	
	**Phenolic acids**	
5.826	Shikimic acid	0.039
13.589	Resveratrol	0.001
15.926	Cinnamic acid	0.005
16.005	3,4-dihydroxycinnamic acid	0.017
22.526	Chlorogenic acid	0.214
22.61	Quinic acid	4.098
24.112	4-hydroxycinnamic acid	0.415
25.972	Coniferin	0.347
26.842	Ferulic acid	0.004
27.991	Piceatannol	0.098
28.192	4-methoxycinnamic acid	0.040
28.829	Sinapic acid	0.294
	**5.571**	
	**Flavonoids**	
11.848	Epicatechin	0.001
14.743	Formononetin	0.003
15.678	Arbutin	0.015
17.184	Catechin	0.001
20.604	Gallocatechin	0.038

33.871	Daidzein	0.023
34.716	Epigallocatechin	0.008
	**0.089**	
	**Alcohols**	
6.067	Butane-2,3-diol	0.157
10.242	Glycerol	2.835
11.168	Phytol	0.878
16.518	1,5-anhydroglucitol	0.087
18.099	Galactitol	0.243
18.099	3-deoxyhexitol	0.243
23.67	Lactitol	1.157
25.286	Galactinol	0.555
26.755	Myo-inositol	4.129
27.991	Piceatannol	0.098
28.302	1-heptanol	0.241
29.9	Dihydrocholesterol	0.118
30.601	2-methylpropan-2-ol	0.490
31.909	Prenol	1.076
35.648	Delta-tocopherol	1.076
	**13.380**	
	**Esters**	
20.537	Glycerol 3-phosphate	0.458
20.963	Inosine-5'-monophosphate	0.213
24.268	Glucaric acid gama-lactone	0.337
27.204	Myristyl myristate	0.415
33.826	Beta-mannosylglycerate	2.373
	**3.796**	
	**Others**	
24.421	D-erythro-sphingosine	2.702
5.068	Methylamine	0.897
10.091	Ethanolamine	0.539
12.958	Putrescine	0.335
25.826	Phytosphingosine	0.123
27.263	Guanine	0.019
15.975	5-hydroxynorvaline	0.009
	...	
